# Measles outbreak investigation in Tocha district, southwestern Ethiopia: an unmatched case–control study

**DOI:** 10.3389/fpubh.2024.1331798

**Published:** 2024-04-10

**Authors:** Simon Fikadu Tefera, Nigatu Admasu, Habtamu Abebe, Gemechu Chemeda Feyisa, Gachana Midaksa

**Affiliations:** ^1^Ethiopian Field Epidemiology Laboratory Training Program (FETP), Jimma University, Jimma, Ethiopia; ^2^Lecturer of Biostatistics, Department of Epidemiology, Jimma University, Jimma, Ethiopia; ^3^Ethiopian Field Epidemiology Laboratory Training Program (EFETP), Jimma University, Jimma, Ethiopia; ^4^School of Public Health, College of Medicine and Health Science, Mizan–Tepi University, Mizan, Ethiopia

**Keywords:** measles, outbreak, Tocha, SWE, Dawuro

## Abstract

**Background:**

Measles continues to be a public health challenge in Ethiopia. Rumors of suspected measles were notified on April 8, 2023 from Tocha district. We conducted an assessment to describe measles outbreak and determine risk factors for measles infection in the Tocha district of the Dawuro zone, Southwest Ethiopia.

**Methods:**

We conducted a 1:2 unmatched case–control studies from April to May 2023. We took all 147 cases registered on line list for descriptive analyses. We used a total of 74 randomly selected cases and 147 controls for case–control part. Any person in Tocha district with laboratory-confirmed measles IgM antibody; or any suspected person epidemiologically linked to confirmed measles cases from March 23 to April 26 2023, were included in the case. Neighborhood who did not fulfill this standard case definition were included in controls. Data were collected using standardized questionnaires deployed on Kobo Collect. Descriptive analyses were conducted using Epi info version 7.2.5.0. The analyses were performed using Statistical Package for Social Science (SPSS) version 26. Binary logistic regression analyses were utilized to select candidate variables. We conducted multiple logistic regression analysis to identify determinants of measles infection at a *p* value ≤0.05 with 95% confidence interval.

**Results:**

The overall attack rate of 22.64/10,000 for general population and 104.59/10,000 among under-five children were attributed to the outbreak with a case fatality rate of 2.72%. Vaccine coverage in the last year and this year were 73.52 and 53.88%, respectively, while vaccine effectiveness in the district was 79%. Poor house ventilation (AOR = 3.540, 95% CI: 1.663–7.535) and having contact history with the case (AOR = 2.528, 95% CI: 1.180–4.557) were positively related to measles infection while being previously vaccinated for measles (AOR = 0.209, 95% CI: 0.180–4.577) reduce risk of measles infections.

**Conclusion:**

The highest attack rate was observed among children under 5 years of age, with a case fatality rate of 2.72%. Vaccination coverage was less than what expected to develop herd immunity. Strategies to increase vaccination coverage and strengthening surveillance systems for rumor identification and early responses to prevent person to person transmission are recommended.

## Introduction

Measles is a highly infectious disease caused by a virus belonging to the paramyxovirus family and is characterized by typical symptoms of fever, a flat red spot rash with raised bumps, cough, runny nose, and pink eye (1, 2). The primary mode of transmission is respiratory secretions expelled by an infected individual (2–4). According to a recent WHO report, an estimated 9.5 million cases and 128,000 people died from measles in 2021, most of whom were under 5 years of age (4). The globe experienced 21 significant and disruptive measles outbreaks in a year, with a large majority of cases from developing countries, including Africa. Ethiopia is among the top five countries affected by measles cases (5, 6).

In developing countries such as Ethiopia, repeated measles outbreaks not only affect infected individuals but also disrupt the country’s health system and socioeconomic status (7). Outbreaks often result in a surge of cases, devastating healthcare facilities and healthcare workers. In addition, measles outbreaks can disrupt and divert attention and resources away from other essential and routine health care, such as maternal and child health (MCH) services (8, 9). Some measures taken to manage outbreaks can also result in increased medical costs, lost productivity due to illness and caretaking responsibilities, and potential school closures to prevent further spread of the virus (10, 11).

As part of the sustainable development goal (SDG), the international community has developed an ambitious goal of eliminating measles by 2030 (12, 13). To achieve this, children 9–59 months old have to develop herd immunity with 95% coverage for two doses of measles-containing virus (MCV). Despite this, many countries have not currently had the impact required to end measles by 2030 (4–6). Only three countries in Africa were able to meet this target by 2020 (6).

With 1,953 cases in 2021, there will be an almost fivefold increase in confirmed measles cases in Ethiopia in 2022 (14). The 2019 Ethiopian Demographic and Mini-Health Survey (EDHS) showed that coverage of first and second doses of the measles vaccine were 59 and 9%, respectively, falling far below the target for measles elimination (15). Along with this, the continued vulnerability of vaccinated children to measles infection has overwhelmed the conditions (16, 17).

Multiple factors may contribute to these problems in Ethiopia, including low population immunity, concurrent epidemics, conflict, forced displacement, and other humanitarian crises that disrupt childhood vaccinations (14). In addition, cultural beliefs, limited awareness, and behavioral factors can act as barriers to seeking early treatment and utilizing other disease management strategies (14, 16, 17). Comprehensive rumor identification and prompt responses are needed to prevent and manage such problems (13, 18).

Rumors of suspected measles cases were reported from the Tocha district of the Dawuro Zone on April 8, 2023. This outbreak investigation was conducted to confirm the rumor and identify potential risk factors for measles infections and implement necessary public preventive and control measures in the affected areas.

## Methods and materials

### Study area and period

The study was conducted in Tocha district from April 10 to May 30, 2023. The district is located in the Dawuro Zone of southwest Ethiopia. The district consists of 15 kebeles, with 14 rural kebeles and 1 urban kebele. From these, the Geda Mela, Bobi, Shechi, and Shada kebeles were located in hard-to-reach areas. The total population of the district was 64,917, with 31,808 males and 33,109 females. Among the total population, 10,917 (15.6%) were children under the age of five, and 2,071 (3.19%) were infants under 1 year old. The district has a total of 13,248 households with an average family size of 4.9. Currently, the district has 15 health posts, 1 primary hospital operated by the government, 3 private clinics, and 1 private pharmacy. The primary hospital in the district offers static immunization services, while health posts provide outreach services (19, 20).

**Figure 1:**
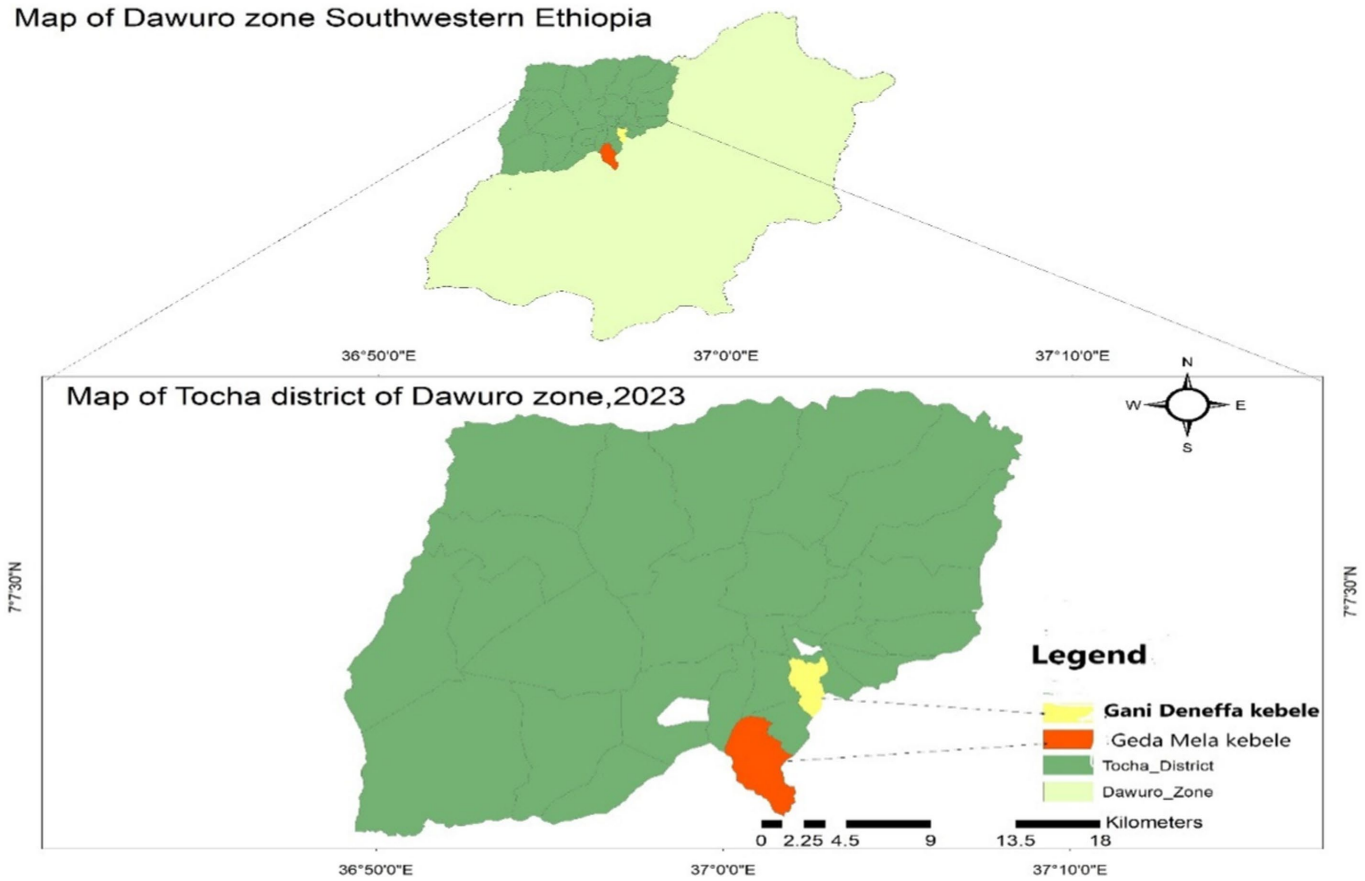
Map of Tocha district Dawuro Zone, Southwest Ethiopia, 2023.

The outbreak primarily occurred in Geda Mela and Gani Denefaa kebeles, situated approximately 12 kilometers and 9 kilometers from Tocha town, respectively (Figure 1).

### Study design

An unmatched case–control study was conducted to describe the measles outbreak and to determine risk factors for measles infection.

### Source and study population

All populations of Tocha district were the source population. People in Tocha district who fulfilled the standard measles case definition and lived in selected kebeles were considered cases. Other people living in similar kebeles but who did not complete the standard case definition of measles were considered controls. Any person in Tocha district with laboratory-confirmed measles IgM antibody; or any suspected person epidemiologically linked to confirmed measles cases from March 23 to April 262,023, were included in the case. Those who had a vaccination history in the past 2 weeks were excluded from cases, provided that they may have developed measles symptoms as a result of an adverse event following immunization. Those who had a previous history of measles infection were excluded from control because they develop immunity against measles.

### Sample size determination

Prior to sample size determination, a sampling frame was established by the following procedure. Immediately after the rumors were reported, five blood samples were obtained from suspected cases and dispatched to the National Public Health Laboratory for measles outbreak confirmation. Accordingly, all five samples tested positive for IgM antibodies.

For the descriptive analysis, we included all 147 registered measles cases on the line lists. However, we determined the sample size for the case–control study using the double population proportion formula assisted by Epi Info software version 7.2.5.0 considering the following assumptions for vaccination status: proportion of control exposed (*p* = 67%, AOR = 2.84), 95% confidence interval, power 80%, and ratio of case to control 1:2 (16, 17). Finally, a large sample size was selected from the three-sample size and found to be 221 for vaccination status. This sample size was finally split into 74 cases and 147 controls. We identified cases that fulfilled the measles case definition by performing active case searches in the affected kebeles and from line lists in health facilities.

### Sampling technique

We selected the two-measles affected kebeles of Tocha district, namely, Geda Mela and Gani Denefaa, in the study. We employed a simple random sampling technique to select cases from line lists of measles cases in the district. Then, we conducted a house-to-house survey to collect data from selected cases in the affected kebeles.

Controls were participants from adjacent neighborhoods of cases who did not fulfill measles case definition, identified from randomly selection. They were all selected from kebeles (the smallest administrative structure) from which cases were selected 42 days after the incubation period of the last case.

### Data collection and procedures

The data were collected using a structured researcher-administered questionnaire adapted from related literature (2, 16, 21), reviewing line lists, patients’ medical records, and observation checklists. The data were collected by two Field Epidemiology residents using the Kobo Collect platform Android version 2022.4.4 under daily supervision.

Observation checklists and recorded secondary data (line lists, patients’ medical records) were used in a descriptive analysis. The measles line lists include the patients’ age and sex, signs and symptoms, date of onset, admission status, vaccination status, laboratory results, treatment outcomes and others. Observation data were used to assess the availability and functionality of refrigerators, vaccine carriers, ice packs, and the overall management of the cold chain system of the district health office.

Questionnaires for case–control studies also collected sociodemographic information, ventilation status, exposure history (contact history with measles cases, travel history and vaccination status), knowledge about measles, and measles clinical features (signs and symptoms).

### Variables and measurements

The outcome variable was measles infection, which was measured based on standard measles case definitions. Accordingly, any suspected person epidemiologically linked to confirmed measles cases who developed fever, maculopapular rash with cough or coryza, conjunctivitis and/or laboratory-confirmed IgM antibody for measles was said to be infected by measles (1).

Independent variables include sociodemographic variables, ventilation status, exposure history (contact history with measles cases, travel history and vaccination status), and knowledge about measles.

#### House ventilation status

Refers to the mechanism to allow exchange of indoor and outdoor air to reduce the risk of measles transmission. We measured the ventilation status using two questions, and the house was said to have good ventilation if it had at least one window that was opened on a daily basis. Otherwise, the house is labeled poorly ventilated.

#### Measles exposure history

The condition that puts a person at risk of measles infection. We measured this condition using three interrelated concepts (contact history with active measles cases, travel history to measles outbreak sites, and vaccination status). Persons who had a history of exposure during these days were said to have a history of contact with active measles cases (1, 2). In addition, a person who traveled to known measles outbreak places was considered to have a travel history (2, 16).

#### Vaccination status

Data about vaccination status were collected both from immunization cards and historical recall of participants. A person was said to be vaccinated if he/she took a minimum of a single dose of measles-containing vaccine (MCV) (2, 16).

#### Knowledge about measles

Knowledge about measles was measured considering most susceptibility status, measles mode of transmission, signs and symptoms, treatment options and prevention methods. Participants were given a score of one (1) for each correct answer and zero otherwise. All correct answers were added together, and the mean score was calculated. Finally, participants who scored greater than the mean score were declared as having good knowledge about measles (22).

#### Suspected measles case definition

Any person with fever, nonvesicular generalized maculopapular rash and cough, coryza or conjunctivitis, or any person in whom the clinician suspects measles (1).

#### Confirmed measles case definition

A suspected case with laboratory confirmed measles IgM antibody or epidemiologically linked to confirmed measles cases in an measles outbreak (1).

#### Epidemiologically linked case

A suspected measles case with no specimen collected for serologic confirmation but linked (in place, person, and time) to a laboratory-confirmed case, that is, living in the same or a neighboring district with a confirmed measles case where there is a likelihood of transmission; onset of rash of the two cases being within 30 days of each other (1).

#### Measles outbreak

Measles outbreak is declared when 3 or more measles infections are laboratory confirmed for measles IgM antibody in a specific district in a month (1).

#### Compatible case

Not epidemiologically linked to laboratory confirmed cases. A suspected case which has not been adequately investigated (1).

#### Measles death

Defined as any death from an illness that occurs in a confirmed measles case or epidemiologically linked case of measles within 1 month of the onset of rash (1).

#### Vaccine effectiveness

Measure the proportion of reduction in measles cases among vaccinated persons. It was determined by calculating the percentage reduction in the incidence rate of measles among unvaccinated individuals and determining the percentage of reduction in risks of cases among vaccinated persons relative to unvaccinated persons. The formula for calculating vaccine effectiveness (VE) is (1-OR) *100. In Ethiopia, this calculation was performed for children aged 9 months to 59 months, provided that the routine measles immunization program starts at 9 months of age and the outbreak in Tocha district primarily affected children under 5 years old (2, 23).

#### Laboratory investigation

We obtained 5 mL of blood samples from five (5) suspected measles cases, using a sterile syringe and needle. Samples were then placed in a sterile tube labeled with the individuals’ details and the date. After separating the serum from the blood, we stored the labeled tubes in a cooler with four ice packs in a vaccine carrier. Subsequently, we sent both the samples and a form detailing the cases to the national health lab for IgM antibody testing. All the five samples tested positive for IgM antibodies.

### Data analysis procedures

The collected data were cleaned and exported to Epinfo version 7.2.5 software for descriptive analyses and Statistical Package for Social Science (SPSS) version 26 for analytical study. Descriptions of cases by person, place and time along the attack rate (AR), case fatality rate (CFR) and vaccine effectiveness were calculated. Bivariate logistic regressions were utilized to select candidate variables with a *p* value of ≤0.25. Multiple logistic regression was performed to identify predictors for measles infection. The level of statistical significance was declared at a *p* value ≤0.05 with a 95% confidence interval (CI).

### Data quality management

The measles case definition was clearly established to ensure that all cases included in the study met the specific criteria outlined, minimizing the risk of misclassification bias. To prevent selection bias and enhance the representativeness of the study findings, both cases and controls were recruited from the same population. Data were collected by Field Epidemiology residents. The supervisor conducted daily checks to ensure the completeness and consistency of the collected data.

## Result

### Review of the outbreak

According to interview result with family members, the index case for the outbreak was a 3-year-old female child who had a travel history to the adjacent Dali town of dawuro zone along with her mother on March 9, 2023. After returning back to Geda Mella kebele, the mother used to bring the child in most of community gathering she participated, like spiritual worshiping conference, market places, and mourning. The case was unvaccinated against measles and started to show rushes and fever on March 23, 2023. The mother gave anti pain bought from rural private drug vender. Later on, the child developed coughing and unable to feed and passed away on April 2, 2023.

**Figure 2:**
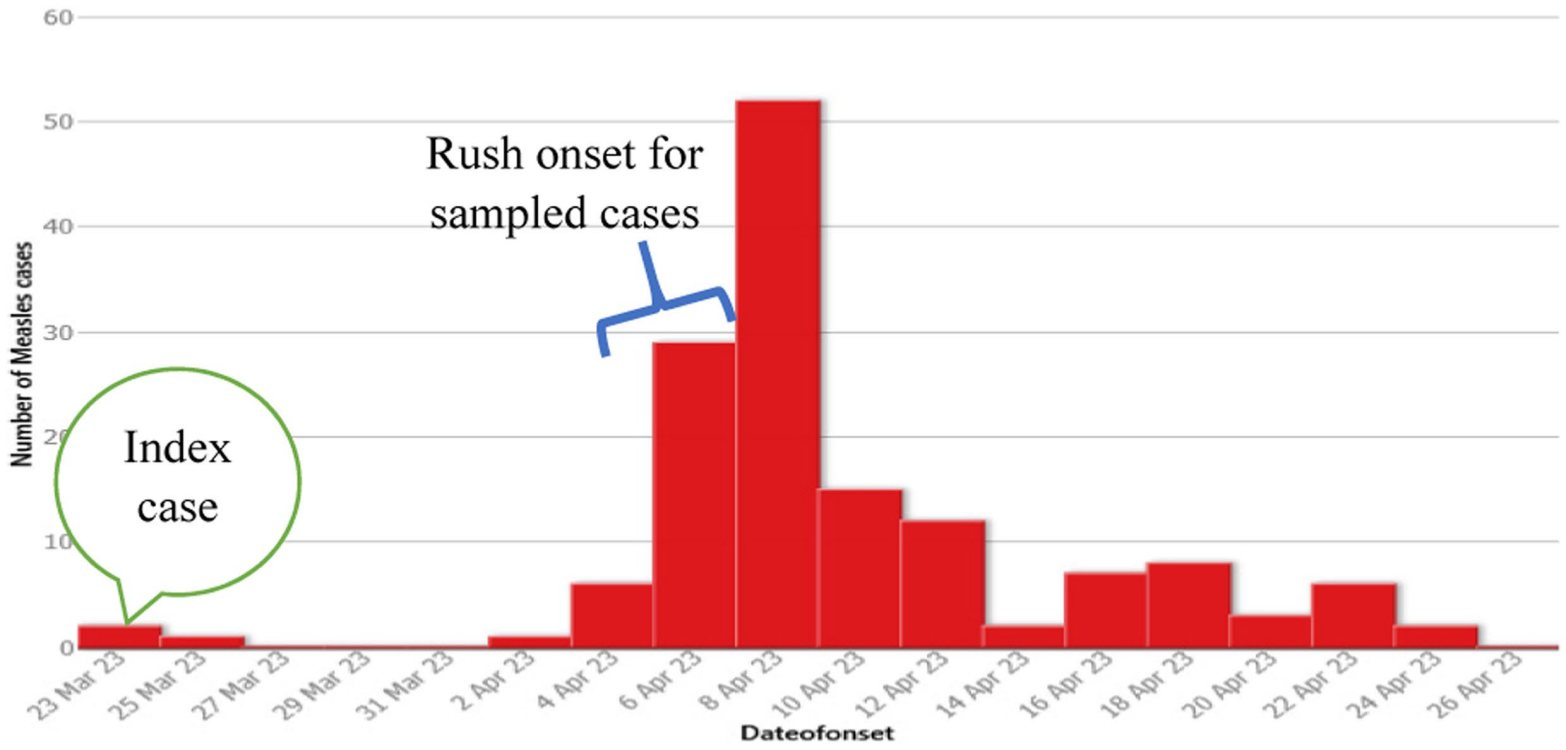
Number of measles cases by date of onset of rash, Tocha district, of the Dawuro Zone from March 23 to April 26, 2023.

During the time, there were many other similar cases in the community that were not brought to health facilities. However, rumors for suspected measles cases were received on April 8, 2023. On the date, the district health office has mobilized to confirm the cases. The zonal health department and regional health bureau took measurements on the next day. A multidisciplinary team engaged on outbreak investigation in the affected kebeles. Blood samples were collected from five suspected cases and sent to the Ethiopian public health institute (EPHI) for laboratory confirmation on April 9, 2023. All of the five suspected cases were found to be positive for measles IgM antibody, which was enough to confirm measles outbreak in Tocha district by April 11, 2023. 3(60%) cases reported to show first rush on April 5 while the rest 2 (40%) on April 6 (Figure 2). The remaining 142 cases were not laboratory tested but, epidemiologically linked with confirmed measles cases. Therefore, the total number of measles cases during the outbreak was 147, with an overall attack rate (AR) of 22.64 per 10,000 population. The outbreak ended with four related deaths with a case fatality rate (CFR) of 2.72%.

### Descriptive epidemiology

#### Cases by time

The Epi curve showed that the first case was registered on March 23, 2023. The peak of the outbreak was observed on April 8, 2023, and the last case occurred on April 26, 2023. It is known that the measles incubation period ranges from 7 to 18 days with an average of 14 days (1). With these data, the possible earliest and last dates of exposure were estimated to be March 16, 2023, and April 7, 2023, respectively. Therefore, the estimated date of exposure for the first case of measles in Torcha district was estimated to be from March 16, 2023, to April 7, 2023. This indicated that the outbreak lasted for two incubation periods (Figure 2).

#### Cases by place

**Figure 3:**
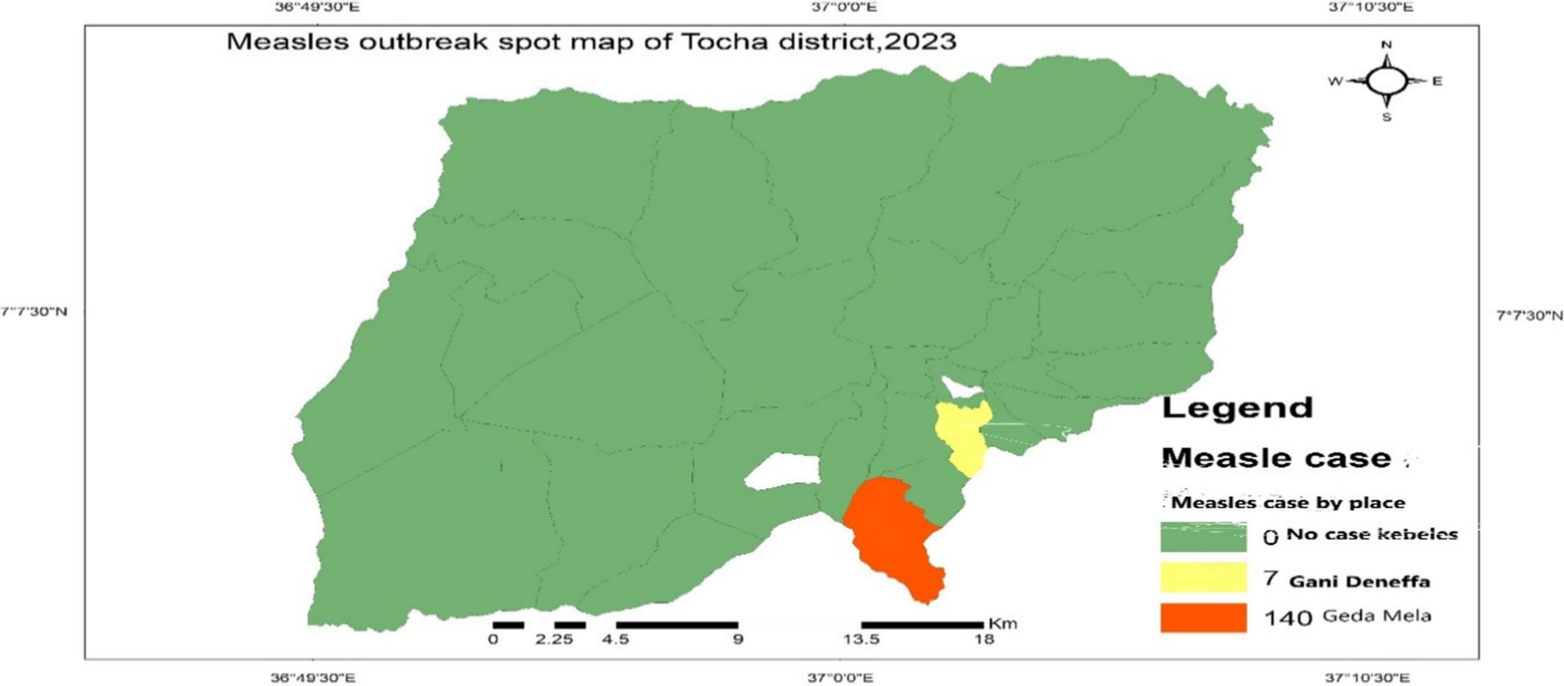
Measles cases by place in Tocha district, 2023.

A total of 147 cases of measles, accompanied by four deaths, were reported from Geda Mela and Gani Denefaa kebeles of Tocha district, with an overall attack rate (AR) of 22.64/10,000 of the population. The majority of cases were reported from Geda Mela kebeles, with an attack rate of 232.37/10,000 population. Geda Mela was the remotest of all other kebeles in the district, situated approximately 12 kilometers from Tocha town in a hard-to-reach area, which makes it difficult to deliver routine immunization services (Figure 3).

#### Cases by person

Among the total measles cases, 74 (50.34%) were males. The attack rates of measles in females and males were almost similar, with 22.05 and 23.26 per 10,000 people in the district, respectively. In addition, the age-specific attack rate for children under 5 years of age was 104.59/10,000 population (Table 1).

**Table 1:** Measles outbreak attack rate in Tocha district, SWE 2023.

Place	Categories by age group	Population at risk	cases	Attack rate per 10,000
Tocha district	District total	64,917	147	22.64
	<5	10,135	106	104.59
	5–14	20,773	39	18.77
	≥15	34,009	2	0.59
Geda Mela	Kebele total	6,025	140	232.37
	<5	940	99	1053.19
	5–14	1925	39	202.60
	≥15	3,160	2	6.33
Gani Deneffa	Kebele total	3,787	7	18.48
	<5	591	7	118.44

### Clinical features of cases

Based on the findings of this study, all 147 cases exhibited signs and symptoms consistent with the standard measles case definition. All cases developed fever and generalized maculopapular rashes. Almost all 145 (98.63%) patients experienced cough. Additionally, patients who developed conjunctivitis and coryza accounted for 12 (8.16%) and 4 (2.72%) patients, respectively. A total of 19 (12.92%) patients were admitted with at least one measles complication. Of these, 6 (4.08%) had developed severe pneumonia, 11 (7.48%) had diarrhea with severe pneumonia, one case experienced severe acute malnutrition, and the remaining case developed tonsillitis abscess with diarrhea.

### Vaccination status of cases

**Figure 4:**
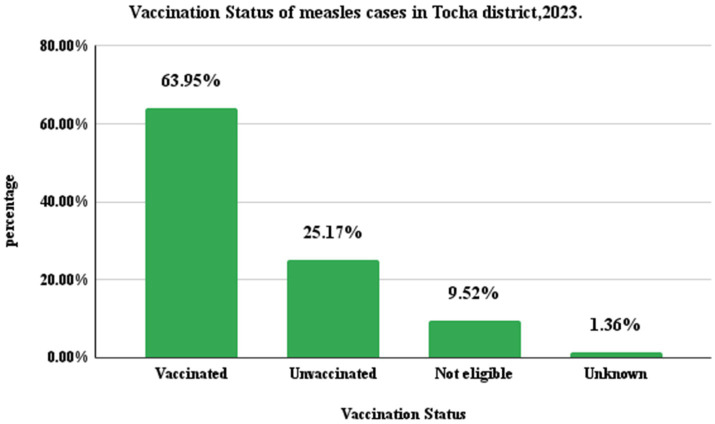
Measles cases by vaccination status in Tocha district of Dawuro Zone, Southwest Ethiopia, 2023.

Among the 147 registered measles cases, almost two-thirds (94, 63.95%) had received at least one dose of measles-containing vaccine (MCV). Approximately one in four cases, 37 (25.17%), were unvaccinated for measles. Fourteen (9.52%) were ineligible for the first dose of MCV, while the vaccination status of two cases, 2 (1.36%), was unknown (Figure 4).

### Measles cases final classifications

**Figure 5:**
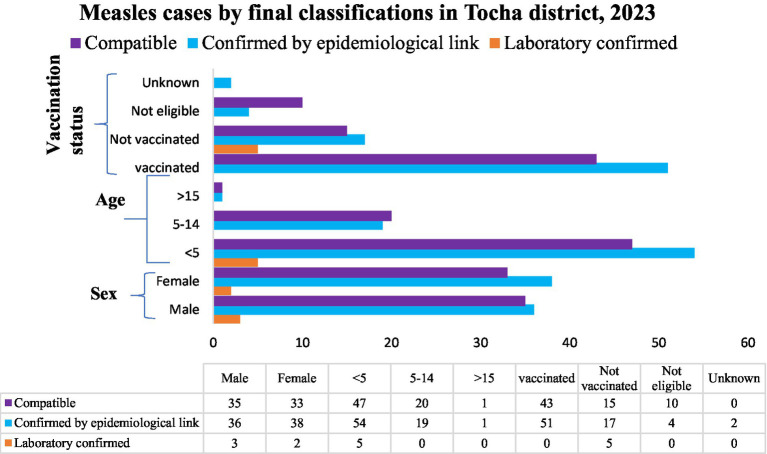
Measles cases status final classification in Tocha district of Dawuro Zone, Southwest Ethiopia, 2023.

Among the registered measles cases, 74 (50.34%) were confirmed by epidemiological link. From these, 38 (51.35%) were female and 54 (72.97%) were children under the age of five. Patients who were not vaccinated for measles accounted for 51(68.92%). From the 68 (46.26%) compatible cases, 35(74.23%) were male. Under five children and those who were not vaccinated for measles accounted for 47 (63.51%) and 43 (58.11%), respectively. In addition, among the five laboratory confirmed cases, 3(60%) were male and all of them were under five children with no history of measles vaccination (Figure 5).

### Vaccination coverage and cold chain management

**Figure 6:**
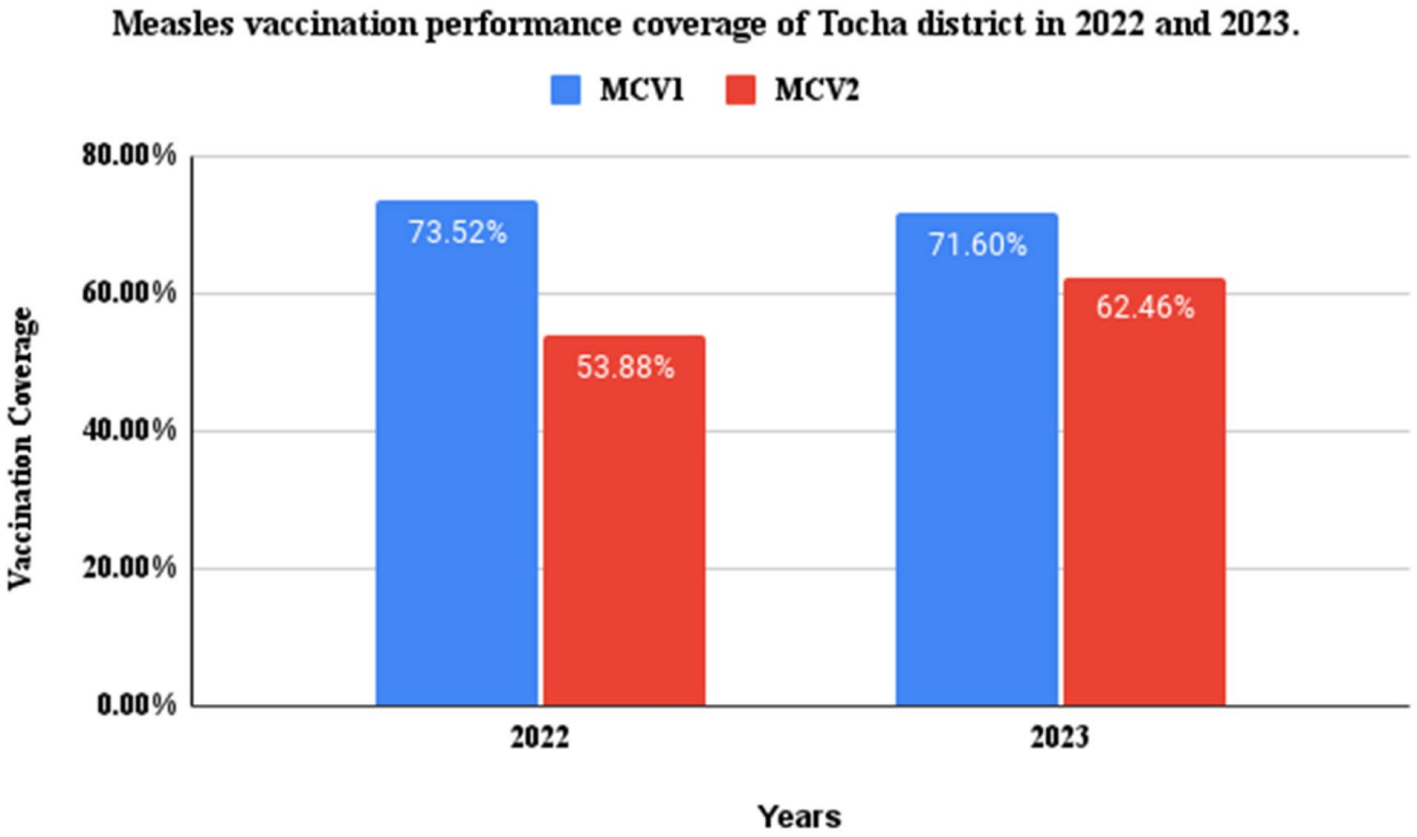
MCV1 and 2 performance coverage of Tocha district, Southwest Ethiopia, in 2022 and 2023.

In the district, the measles vaccination coverage in 2022 for MCV1 and MCV2 was 1,497 (73.52%) and 1,097 (53.88%), respectively. In 2023, the measles vaccination coverage was 1,112 (71.60%) for MCV1 and 970 (62.46%) for MCV2. From these data, the calculated dropout rates of measles vaccination in 2022 and 2023 were 26.72 and 12.76%, respectively. Only Tocha Primary Hospital and six health posts in the district had a refrigerator. From these, two refrigerators were not functional, including that of Geda Mella Kebele. All the assessed health facilities had an adequate supply of vaccine carriers and ice packs. However, power interruptions were common in the district, so the refrigerators were supplied with kerosene and solar energy (Figure 6).

### Analytical epidemiology

#### Socioeconomic characteristics of participants

Two hundred twenty-one (74 cases and 147 controls) participants were involved in this study, and all of them were from the Dawuro ethnic background. The mean age of the participants was 3.60 (SD =1.62) for the case group and 3.12 (SD = 1.32) for the control group at the time of survey. Regarding gender, 46 (62.16%) cases and 81 (55.10%) controls were male. The majority of mothers/caregivers in both groups followed the Protestant religion, with 62 (83.78%) of the cases and 123 (83.67%) controls. Approximately two-thirds of caregivers, 53 (71.62%) of cases and 95 (64.35%) of controls, had engaged in farming. Caregivers who did not attend school accounted for 42 (56.77%) cases and 73 (49.67%) controls. Regarding housing conditions, 45 (60.8%) cases and 127 (83.4%) controls lived in houses with good ventilation status.

Caregivers who had good knowledge about measles accounted for 35 (47.3%) cases and 100 (68%) controls. A higher percentage of cases (47, 63.5%) than controls (62, 41.1%) had a contact history. Regarding vaccination status, approximately 30 (40.54%) of the cases and 117 (79.59%) of the controls were vaccinated (Table 2).

**Table 2:** Sociodemographic characteristics in cases and controls of measles and the respondents in Tocha district, 2023.

Variables	Categories	Case	Control	*X*^2^ test *p* value
Gender of the Child	Male	46(62.16%)	81(55.1%)	0.316
	Female	28(37.84%)	66(44.9%)	
Occupation of the mother/caregiver	Farmer	53(71.62%)	95(64.63%)	0.687
	House wife	17(22.98%)	45(30.61%)	
	Student	2(2.7%)	4(2.72%)	
	Public servant	2(2.7%)	3(2.04%)	
Religion of the mother/caregivers	Protestant	62(83.78%)	123(83.67%)	0.983
	Orthodox	12(16.22%)	24(16.33%)	
Educational level of the mother/caregiver	Unable Read and write	42(56.77%)	73(49.67%)	0.019
	Read and write	10(13.51%)	45(30.61)	
	Elementary	16(21.62%)	25(17%)	
	Secondary and above	6(8.10%)	4(2.72%)	
Housing condition	Ventilated	45(60.8)	127(86.3)	0.000
	Not Ventilated	29(39.2)	20(13.7)	
Knowledge about measles	Good	35(47.3)	100(68)	0.003
	Poor	39(52.7)	47(32)	
Contact history	Yes	47(63.5)	62(41.1)	0.003
	No	27(36.5)	85(57.9)	
Travel history	Yes	33(44.6)	51(34.7)	0.152
	No	41(54.4)	96(65.3)	
Vaccination status	Vaccinated	30(40.54)	117(79.59)	0.000
	Unvaccinated	44(59.46)	30(20.41)	

### Vaccine effectiveness

In this measles outbreak investigation, vaccine effectiveness was found to be 79%.

### Factors associated with measles infections

In the bivariate binary logistic regression, family size, vaccination status, contact history with suspected or confirmed measles cases, age of the child, knowledge about measles transmissions, travel history and house ventilation status were identified as significant predictors of measles infection at *p* values less than 0.25. After controlling for possible confounders in multivariate logistic regression, however, only vaccination status, contact history and house ventilation status continued to have a statistical association with measles infection. Accordingly, being previously vaccinated for measles reduce risk of measles infections by 79% compared with unvaccinated people (AOR = 0.209, 95% CI: 0.180–4.577).

On the other hand, the odds of contracting measles are increased by 3.540 factors for people living in poorly ventilated houses compared to those who live in ventilated houses (AOR = 3.540, 95% CI: 1.663–7.535). Similarly, people who had contact history with known or suspected measles cases were 2.528 times more likely to be infected with measles than their counterparts (AOR = 2.528, 95% CI: 1.180–4.557) (Table 3).

**Table 3:** Bivariate and multivariable logistic regression analysis of measles infection in Tocha district, Southwest Ethiopia, 2023.

Variables	categories	Case status		COR 95% C. I	AOR 95% C. I	*p*. value
		Case (*N* %)	Control (*N* %)			
Family size	<5	33(44.6)	82(55.78)	1	1	
	>5	41(55.4)	65(44.22)	1.567(0.893,2.750)		0.077
Vaccination status	Vaccinated	30(40.54)	117(79.59)	0.175(0.095,0.323)	0.209(0.180,4.577)	0.000*
	Unvaccinated	44(59.46)	30(20.41)	1	1	
Age of the child	<1	15(20.27)	18(12.24)	1	1	
	1–4	59(74.73)	129(87.76)	0.549(0.259,1.163)		0.201
Knowledge about measles	Good	35(47.3)	100(68)	1	1	
	Poor	39(52.7)	47(32)	1.909(1.077,3.387)		0.307
Housing condition	Ventilated	45(60.8)	127(86.3)	1	1	
	poorly ventilated	29(39.2)	20(13.7)	4.092(2.108,7.945)	3.540(1.663,7.535)	0.001*
Contact history	Yes	47(63.5)	62(41.1)	2.386(1.342,4.243)	2.528(1.180,4.557)	0.015*
	No	27(36.5)	85(57.9)	1	1	
Travel history	Yes	33(44.6)	51(34.7)	1.515(0.856,2.680)	1.327(0.678,2.596)	0.408
	No	41(54.4)	96(65.3)	1	1	

^*^Significantly associated variables *p.* value < 0.05.

## Discussion

The World Health Organization and other partners strive to eliminate measles by 2030, with 95% coverage of measles vaccination among children 9–24 months old (13). Outbreak investigation and timely response are other pillars among the core components of measles elimination strategies worldwide (24, 25). The aim of this study was to describe the magnitudes of measles cases and determinants of measles infection that contributed to measles outbreaks in the Tocha district of the Dawuro zone.

In the study area, the overall measles AR was 22.64 per 10,000 population. The AR was higher among under-five children, with 104.59 cases per 10,000 population. Similarly, the AR was higher among residents of Geda Mela kebele, with 232.37 per 10,000 population. This finding was consistent with a similar study from the Garda Marta District of the Gofa zone (16). However, it is higher than the measles outbreak investigation findings from Yemen (26), Guradamole District of Bale Zone (17), Basso Liben District of Amhara region (27) and Nunukumba District, East Wollega Zone (28). This might be due to the large number of unvaccinated children in Tocha district, which made the under-five children more susceptible to measles infection (16, 17).

In addition, the poor cold chain management and low vaccination coverage of the district in general and Geda Mella kebele in particular could have contributed to the outbreak. Although all health posts had icepacks during the survey, only one-third of them had refrigerators. These findings may imply the need to find a lasting strategy for appropriate cold chain management in the districtSimilarly, the case fatality rate (CFR) of measles infection in the district was 2.72%. The findings of our study were lower than those of studies from the Gurada Mole district of the Bale Zone (17) and the expected CFR during measles outbreaks (1). The relatively lower CFR observed in this study may be attributed to unregistered deaths at the community level. However, this finding is higher compared to the study conducted in South Sudan (29), Garda Marta district of Gofa zone (16), and Ginnir district of Bale zone (21). This difference could be attributed to a delayed outbreak response, as the first response was initiated after the index cases had passed away. This may call for strengthening the surveillance system of Southwest Ethiopia in general and the Dawuro zone and Tocha district in particular.

In line with these findings, the district measles vaccination coverage of MCV1 and MCV2 over the past two consecutive years (2022–2023) was 73.52 and 53.88%, respectively. In addition, the district measles vaccination dropout rates over these 2 years were 26.72 and 12.76%, respectively. These were far below the target of WHO measles immunization coverage as the strategy to eliminate measles by 2030 (13, 24).

The difficult topography of the local landscape and the fact that the health post was situated very far from densely populated residences in hard-to-reach areas might also enable health extension workers to trace and vaccinate unvaccinated children in the community. One HEW serves more than 6000 people in highly affected kebeles, such as Geda Mella, which may also affect the quality and equity of vaccination services in the district. The Ethiopian health indicator 2021 suggested that one health post should have to serve 5000 people (30).

Our multivariable analysis demonstrated that being previously vaccinated for measles had reduced risk of acquiring measles infection by 79%. This finding aligns with another measles outbreak investigation from Garda Marta, Sinana district of Oromia and Sekota Zuria district of Amhara regions (16, 31, 32). This may call for strengthening the vaccination strategy to decrease the risk of being at risk of measles infection among 9–59-month age groups. However, this was lower compared with similar study findings from the Ginnir districts of the Bale zone (21). It was also below the WHO-confirmed measles vaccine protective ability that could be achieved among children who received MCV1 (2, 24). This might be attributed by the poor cold chain management of the district in general and Geda Mella kebele in particular that made even the vaccinated children susceptible to measles infection. Although all health posts had icepacks during the survey, only one-third of them had refrigerators. These findings may imply the need to find a lasting strategy for appropriate cold chain management in the district.

Additionally, we found that individuals who had contact history with measles cases had 2.528 times the odds of acquiring measles infection compared to their counterparts. This finding is consistent with a study conducted in the rural district of Ethiopia, the Garda Marta district of Gofa zone and Yemen (26, 16, 31). In fact, measles-infected persons are highly likely to transmit the virus from 4 days prior to the onset of the rash to 4 days after the rash erupts (1). Public health surveillance systems should be strengthened in early rumor identification and management and early response and mitigation to control diseases in elimination phases, such as measles.

Finally, the likelihood of contracting measles is increased by 3.540 factors for people living in poorly ventilated houses compared to those who live in ventilated houses. A systematic review and study findings from America also confirmed the existence of an association between poor house ventilation and the spread of airborne infections, such as measles (33, 34). This demonstrates the need to take measures to reduce overload and increase ventilation in suspected measles cases.

### Strengths and limitations of the study

Due to the nature of case control study design, it was difficult to control for unobserved/observed characteristics among control/case. We used a 1:2 case control to increase probability of getting sufficient number of controls with the same characteristics as cases. In addition, the fact that the care givers may not recognize exact information about some data like vaccination history, these may led to recall bias. However, we tried to manage this by observing vaccination card for those who could show it. Furthermore, we could not get data over the past 5 years to calculate vaccination coverage over 5 years and measles antibody titers by age group.

## Conclusion

The highest AR was noticed among children under-5 years of age, with a CFR of 2.72%. Vaccination coverage and VE among children 9–59 months old were less than expected to develop herd immunity. Strategies to increase vaccination coverage and strengthen surveillance systems for rumor identification and early responses to control the spread of infections via contact and poor ventilation of houses are recommended.

### Public health actions/interventions

In response to the measles outbreak, we took various measures to ensure effective treatment and control of the disease in collaboration with the Southwest Ethiopia People Regional Health Bureau, the Public Health Institute, and the Tocha District Health Office of the Dawuro Zone. Measles cases with complications were treated with antibiotics, oral rehydration salts and supplementary feedings as necessary.

Following confirmation of the outbreak, a catch-up vaccination campaign was conducted, along with a joint nutritional screening. A total of 877 children were vaccinated during the campaign. Additionally, all 6- to 59-month-old children were supplemented with vitamin A to boost the immune response and prevent further complications. We also conducted capacity building on job orientation for health workers and HEWs on measles case definitions. Health education was carried out in schools and churches, with key messages prepared on measles prevention and control measures.

## Data availability statement

The original contributions presented in the study are included in the article/supplementary material, further inquiries can be directed to the corresponding author.

## Ethics statement

The studies involving humans were approved by Southwest People Regional Health Bureau Public Health Institute Institutional Review Board. The studies were conducted in accordance with the local legislation and institutional requirements. The human samples used in this study were acquired from primarily isolated as part of your previous study for which ethical approval was obtained. Written informed consent for participation was not required from the participants or the participants’ legal guardians/next of kin in accordance with the national legislation and institutional requirements. Written informed consent was obtained from the individual(s), and minor(s)’ legal guardian/next of kin, for the publication of any potentially identifiable images or data included in this article.

## Author contributions

ST: Conceptualization, Formal analysis, Investigation, Methodology, Supervision, Validation, Writing – original draft, Writing – review & editing. NA: Conceptualization, Investigation, Writing – review & editing. HA: Conceptualization, Data curation, Methodology, Writing – review & editing. GF: Conceptualization, Data curation, Supervision, Validation, Writing – review & editing. GM: Data curation, Methodology, Software, Validation, Writing – review & editing.
